# Esthetic Rehabilitation of Partially Edentulous Ridge With Horizontal Bone Augmentation Using the Sausage Technique: A Report of Two Cases

**DOI:** 10.7759/cureus.56015

**Published:** 2024-03-12

**Authors:** Grazina Fernandes, Meena Aras, Vidya Chitre, Ashwin Mysore, Saurabh Kamat

**Affiliations:** 1 Department of Prosthodontics and Crown and Bridge, Goa Dental College and Hospital, Panaji, IND; 2 Department of Oral and Maxillofacial Surgery, Goa Dental College and Hospital, Panaji, IND

**Keywords:** prosthetic rehabilitation, bone graft, implants, sausage technique, esthetics, ridge augmentation

## Abstract

Bone augmentation techniques have been used in atrophic ridges to attain appropriate bone volume and enable dental implant insertion. By reducing the need for autogenous bone and decreasing morbidity at the donor sites, the use of bone substitutes has improved patient comfort and satisfaction. One of the major challenges in implant dentistry is achieving an optimal esthetic result with implant-supported crowns in the esthetic zone. Multiple prosthetic and surgical aspects need to be carefully planned and executed to achieve the final esthetic result. This is a report describing bone augmentation using the sausage technique and subsequent prosthetic rehabilitation in two cases.

## Introduction

Dental implants have become a commonly accepted method of replacing missing teeth. An ideal foundation for delivering an implant-supported prosthesis is the osseointegrated implants. Adequate bone volume is required for implant placement. The resorption of the alveolar ridge is a result of tooth extraction or loss. During the first year following tooth extraction, the alveolar ridge decreases rapidly in width and height. A bone defect is created as a result of this. Dental trauma, accidents, pathologic resorption, periodontal disease, and iatrogenic factors can all cause these defects [1,2].

Several methods have been proposed to increase the horizontal width or vertical height of the alveolar bone. One of these, named the sausage approach was developed by Istvan Urban, which includes placing the bone graft material toward the crest and fixing a collagen membrane over it with titanium pins. As there is sufficient graft material filled inside the fixed membrane, it produces a balloon effect. The graft material is pushed in the crestal direction, producing tension in the membrane [3-5].

This is a report of two cases of deficient implant sites in the maxilla and mandible, respectively, which were rehabilitated using the sausage technique.

## Case presentation

Case 1

**Figure 1:**
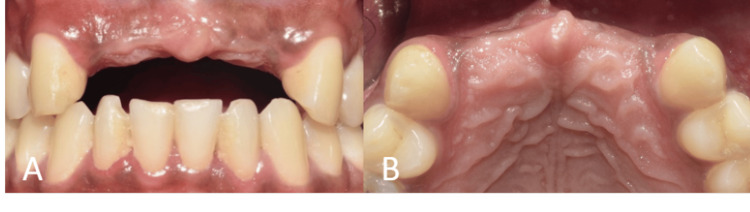
Preoperative intraoral view showing missing maxillary incisors. (A) Frontal view on maximum intercuspation showing the edentulous maxillary anterior region in relation to teeth no. 11, 12, 21, and 22; (B) occlusal view showing the edentulous maxillary anterior ridge.

**Figure 2:**
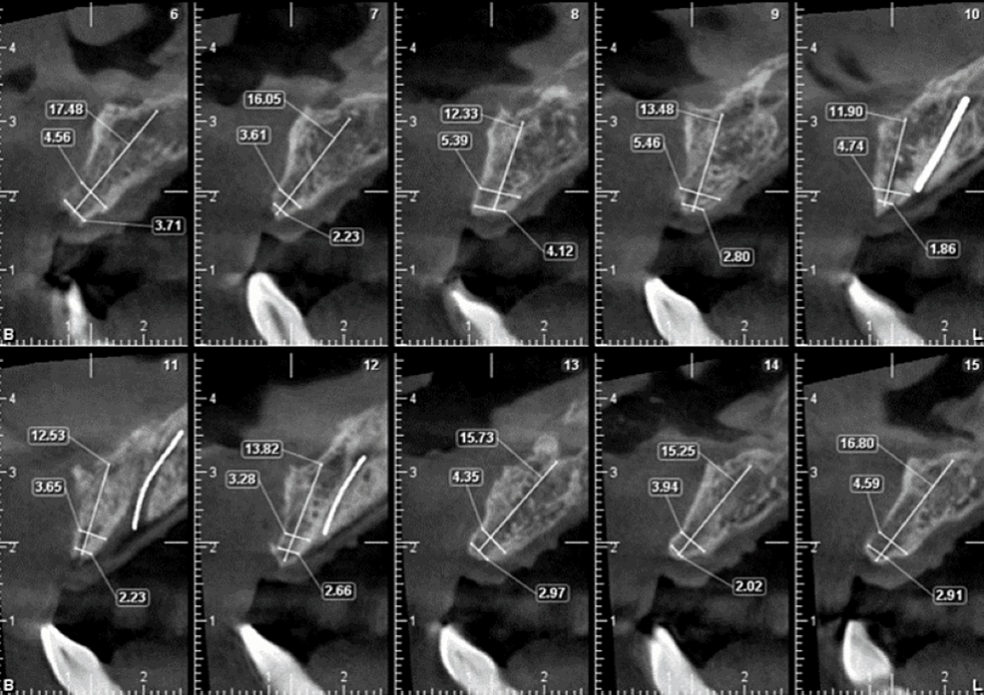
Preoperative CBCT of anterior maxilla showing bone width at different sections. CBCT, cone beam computed tomography

A healthy, 30-year-old male patient presented with missing maxillary incisors. Clinical examination revealed Seibert's class I defect (Figure 1). A cone beam computed tomography (CBCT) scan was performed, which confirmed horizontal alveolar bone resorption at the site (Figure 2). Rehabilitation of the missing incisors with implants and bone augmentation was decided upon as the treatment of choice.

**Figure 3:**
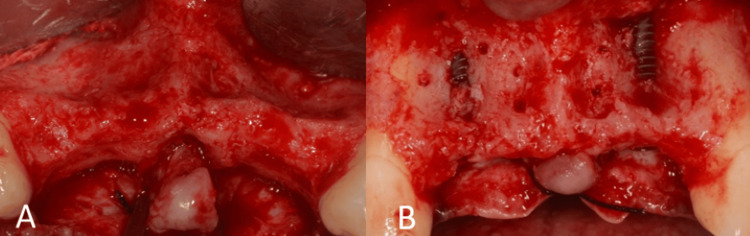
Surgical procedure and implant placement. (A) Flap elevation showing the labial defect; (B) implant placement in relation to teeth no. 12 and 22.

Before surgery, the patient was instructed to rinse with 0.2% chlorhexidine for one minute. A 5% povidone-iodine solution was used to disinfect the perioral skin. The surgical procedure was performed under local anesthesia (2% lignocaine with 1:80,000 adrenaline). To gain access, a crestal incision was made, and intrasulcular incisions were made on the labial surfaces of adjacent teeth in addition to releasing incisions. A full-thickness mucoperiosteal flap was raised (Figure 3).

**Figure 4:**
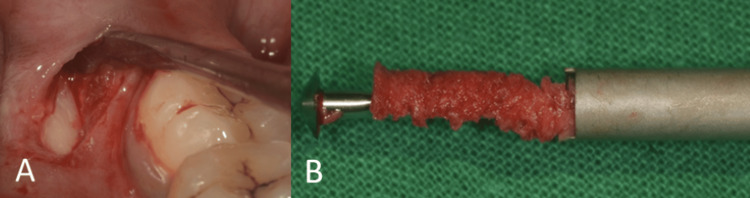
Harvesting autogenous bone. (A) Flap elevation to harvest autogenous bone from the ramus of the mandible; (B) bone scraper showing autogenous bone.

Conventional implant (3.5 mm x 13 mm, Osstem Implant TS III, Seoul, Korea) placement was performed at the lateral incisor sites. In addition to implant placement, deproteinized bovine bone xenograft particles (Bio-Oss; small granules, Geistlich Pharma, Wolhusen, Switzerland) and resorbable collagen membrane (Bio-Gide, Geistlich Pharma) were used for bone augmentation. Autogenous bone scrapes were harvested from the ramus of the mandible using Micross Bone Scraper (Meta, Reggio Emilia, Italy) (Figure 4).

**Figure 5:**
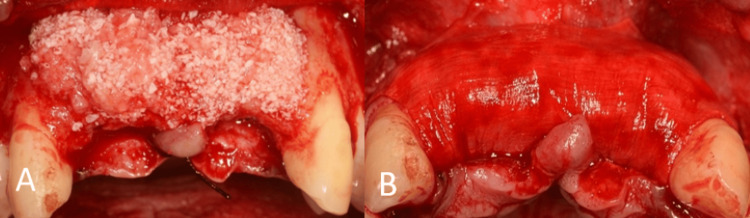
Surgical procedure. (A) Placement of the mixture of autogenous and xenogeneic bone graft material; (B) fixation of the Bio-Gide membrane using titanium pins.

The labial aspect of the defect was filled with an autogenous graft over the exposed implant surface followed by a combination of autogenous and xenogeneic bone grafts, and the membrane was then secured to the labial aspect using titanium pins. The membrane was stretched to produce a balloon effect. The flap was then sutured. The patient was given postoperative instructions and discharged (Figure 5).

**Figure 6:**
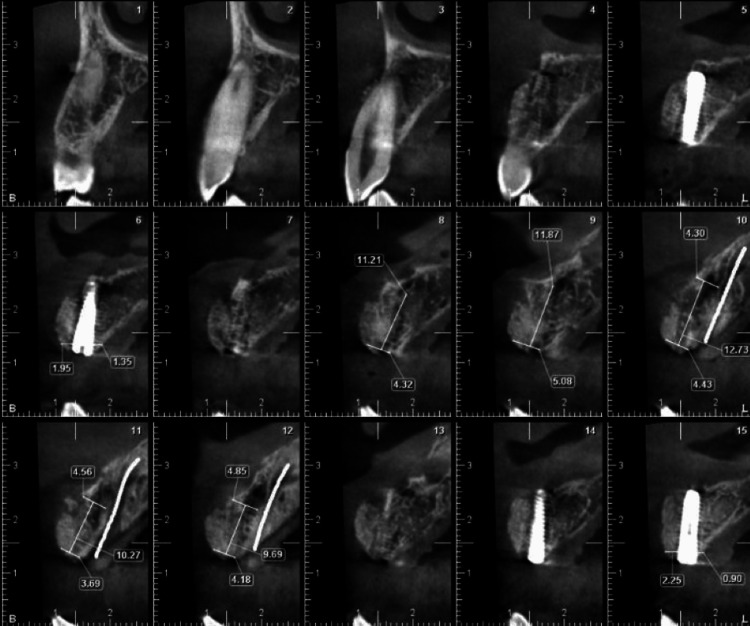
Postoperative CBCT of anterior maxilla showing the regenerated site and implants. CBCT, cone beam computed tomography

**Figure 7:**
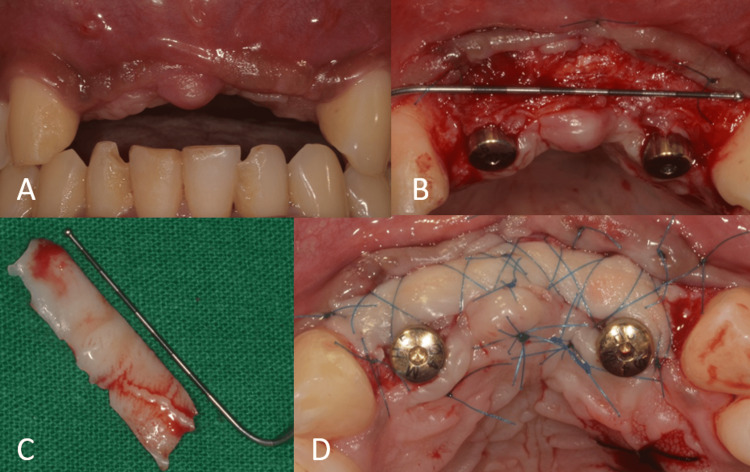
Surgical procedure. (A) Frontal view showing inadequate attached gingiva; (B) flap elevation showing the measurement for grafting the recipient site; (C) free gingival graft harvested from the palate; and (D) placement of the free gingival graft.

The site presented a horizontal bone gain of 5 mm on CBCT after six months of healing (Figure 6). Second-stage surgery was performed with free gingival grafting in the anterior labial region due to inadequate attached gingiva (Figure 7).

**Figure 8:**
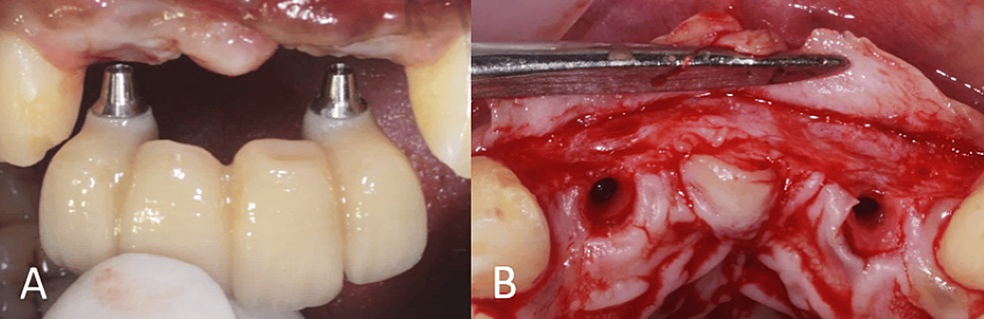
Surgical procedure. (A) Frontal view of the interim prosthesis; (B) occlusal view showing the crestal incision before the placement of interim prosthesis to enhance the gingival contour.

One month after soft-tissue grafting, an interim prosthesis was inserted using a crestal incision to enhance and sculpt the gingival contour (Figure 8).

**Figure 9:**
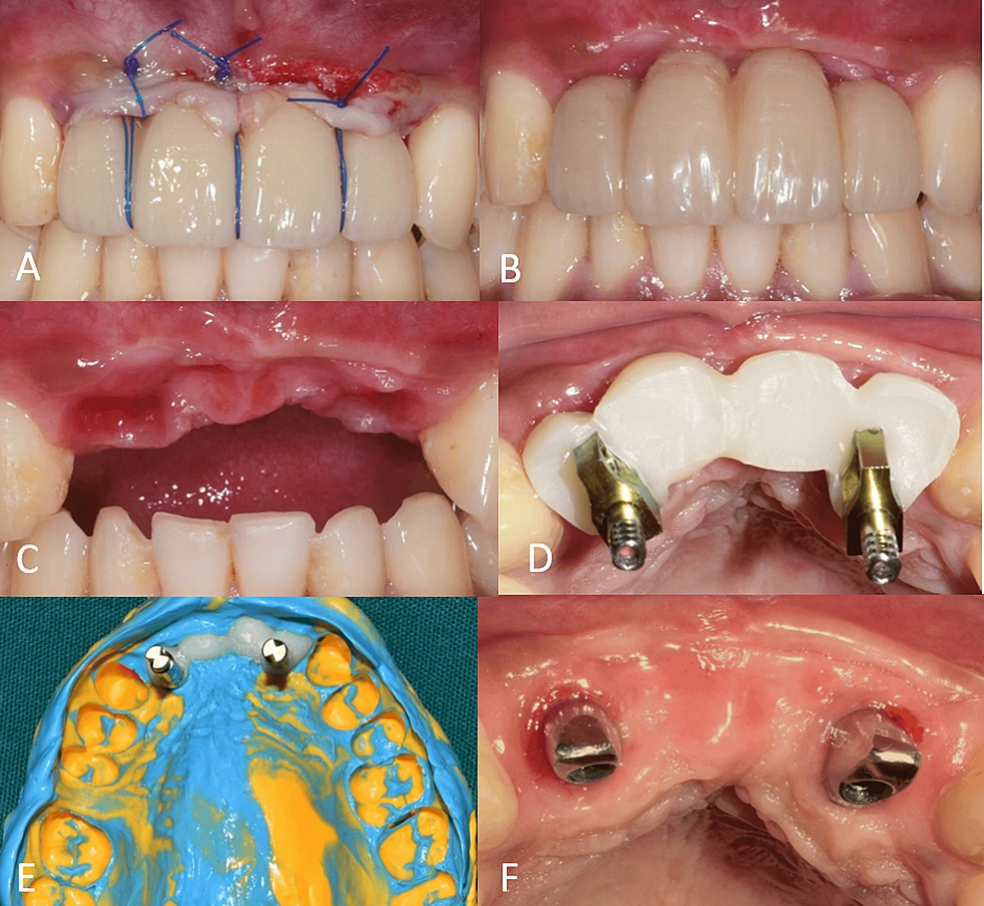
Interim and final prosthesis procedure. (A) Frontal view of the interim prosthesis after suturing; (B) frontal view of the interim prosthesis after recontouring with composite; (C) frontal view of the molded gingiva; (D) occlusal view of the customized open-tray impression copings; (E) open-tray implant impression; and (F) occlusal view showing customized abutments.

**Figure 10:**
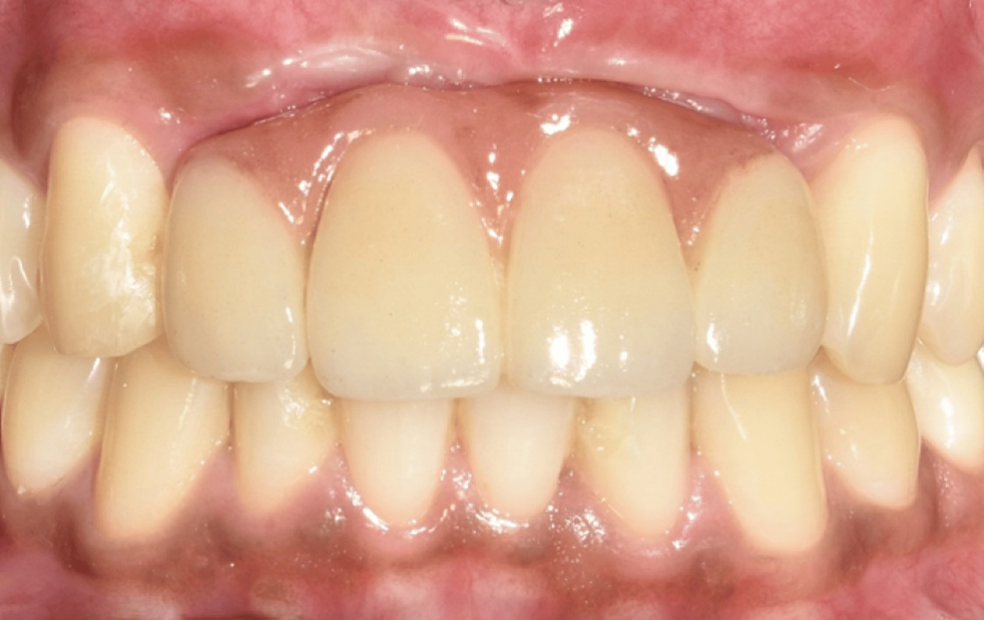
Final implant prosthesis.

The gingiva in the cervical region of the interim prosthesis was molded by adding composite to the interim prosthesis (Figures 9A-9B). Open-tray implant impression was made after replicating the contours of the interim prosthesis onto impression copings with composite (Figures 9D-9E). The final prosthesis was cemented over customized abutments (Figures 9F, 10).

Case 2

**Figure 11:**
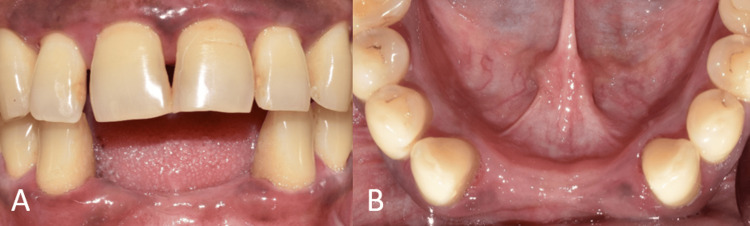
Preoperative intraoral view showing missing mandibular incisors. (A) Frontal view at maximum intercuspation, depicting the edentulous mandibular anterior region in relation to teeth no. 31, 32, 41, and 42; (B) occlusal view showing the edentulous mandibular anterior ridge

**Figure 12:**
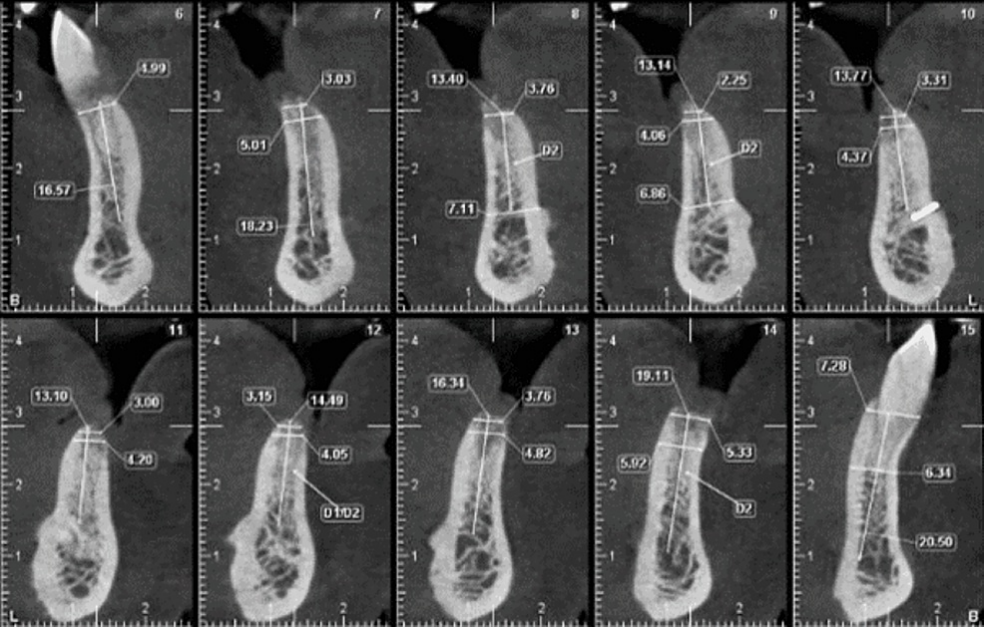
Preoperative CBCT of the anterior mandible showing the bone width at different sections. CBCT, cone beam computed tomography

A 52-year-old male patient presented with missing mandibular incisors. Clinical examination revealed a Seibert's class I defect (Figure 11). The CBCT revealed vertical and horizontal bone resorption at the site (Figure 12). The insertion of implants in the appropriate three-dimensional (3D) location was ruled out unless a bone reconstruction was performed prior.

It was decided that the treatment of choice in this case would be to rehabilitate the missing incisors with implants with prior bone augmentation.

**Figure 13:**
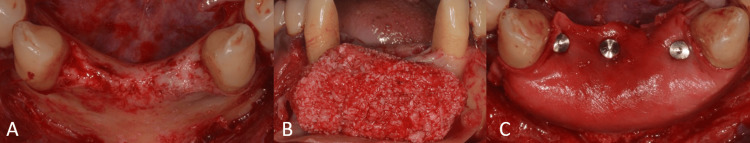
Surgical procedure. (A) Flap elevation showing the labial defect; (B) placement of Bio-Oss bone graft material; and (C) fixation of the Bio-Gide membrane using titanium pins

The patient was given instructions to rinse with 0.2% chlorhexidine for one minute before the procedure. The perioral skin was disinfected with a 5% povidone-iodine solution. Local anesthetic (2% lignocaine with 1:80,000 adrenaline) was used during surgery. In addition to releasing incisions, crestal and intrasulcular incisions were made on the labial surfaces of adjacent teeth to get access. A mucoperiosteal flap of full thickness was raised (Figure 13A).

A deproteinized bovine bone xenograft (Bio-Oss; small granules, Geistlich Pharma) was used to fill the defect's labial aspect. A resorbable collagen membrane (Bio-Gide, Geistlich Pharma) was used to cover the graft. Titanium pins (3 mm Metapin, Meta) were then used to secure the membrane to the labial aspect. The balloon effect was created by stretching the membrane and securing it (Figures 13B-13C). The flap was sutured and primary closure was obtained. Postoperative instructions were provided to the patient.

**Figure 14:**
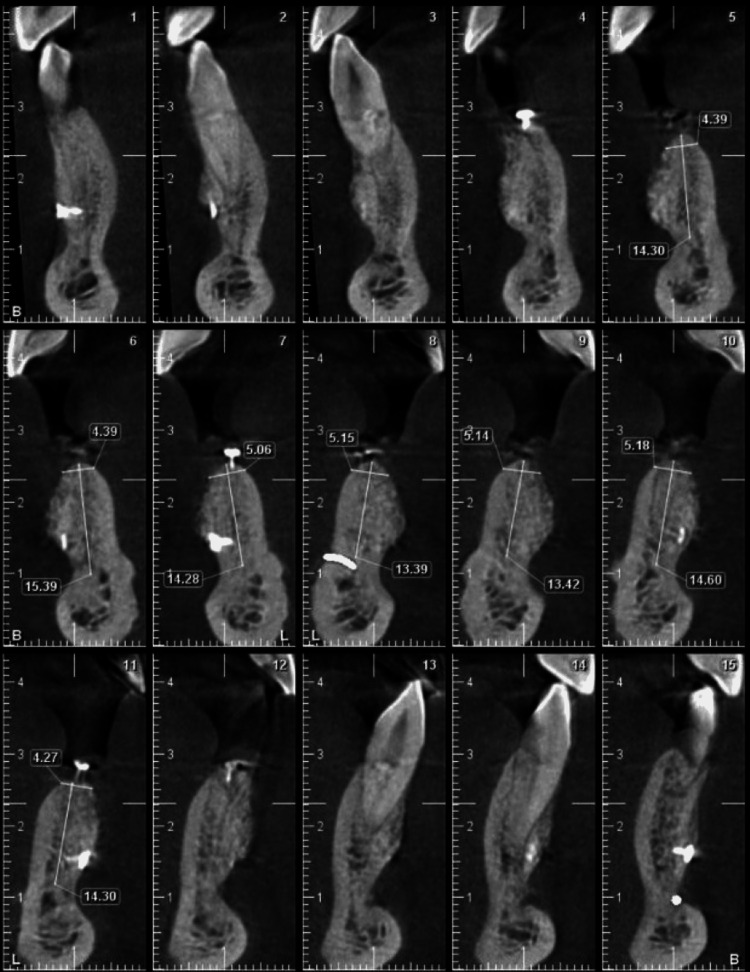
Postoperative CBCT of the anterior mandible showing the regenerated site. CBCT, cone beam computed tomography

**Figure 15:**
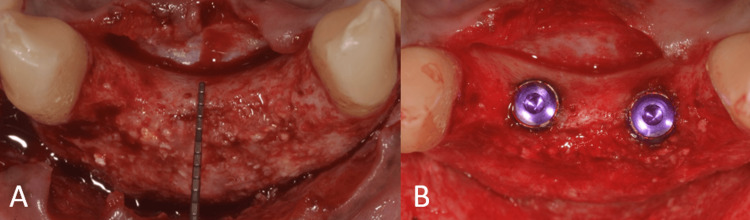
Surgical procedure. (A) Flap elevation showing the regenerated site; (B) implant placement in relation to teeth no. 32 and 42.

After six months of healing, the site was reopened for implant placement. The bone demonstrated a horizontal width of 5 mm (Figure 14). Two 3.5 mm x 11 mm (Osstem Implant TS III) were placed bilaterally in the lateral incisor regions (Figure 15).

**Figure 16:**
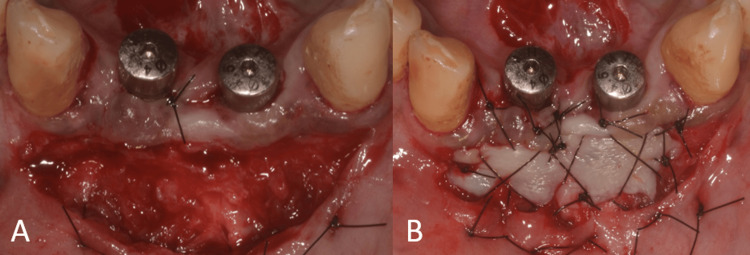
Soft-tissue grafting. (A) Flap elevation for the placement of free gingival graft; (B) placement of the free gingival graft.

**Figure 17:**
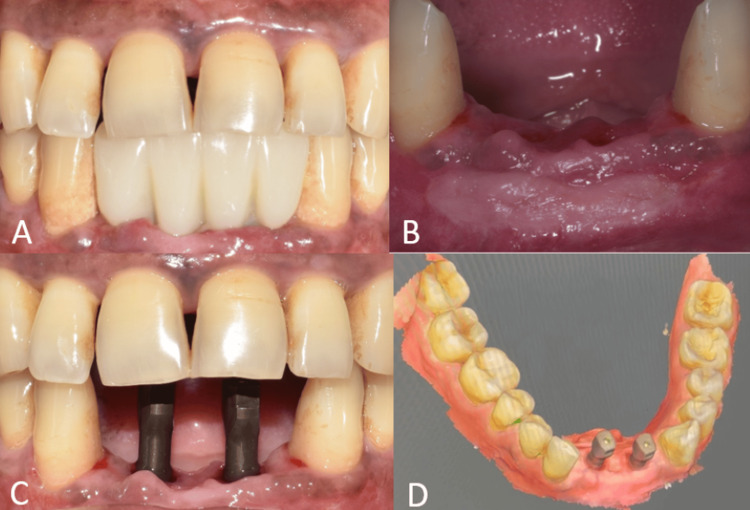
Interim and final prosthesis procedures. (A) Frontal view of the interim prosthesis; (B) frontal view of the molded gingiva; (C) frontal view of the mini-scan bodies; and (D) digital impression to fabricate the prosthesis

**Figure 18:**
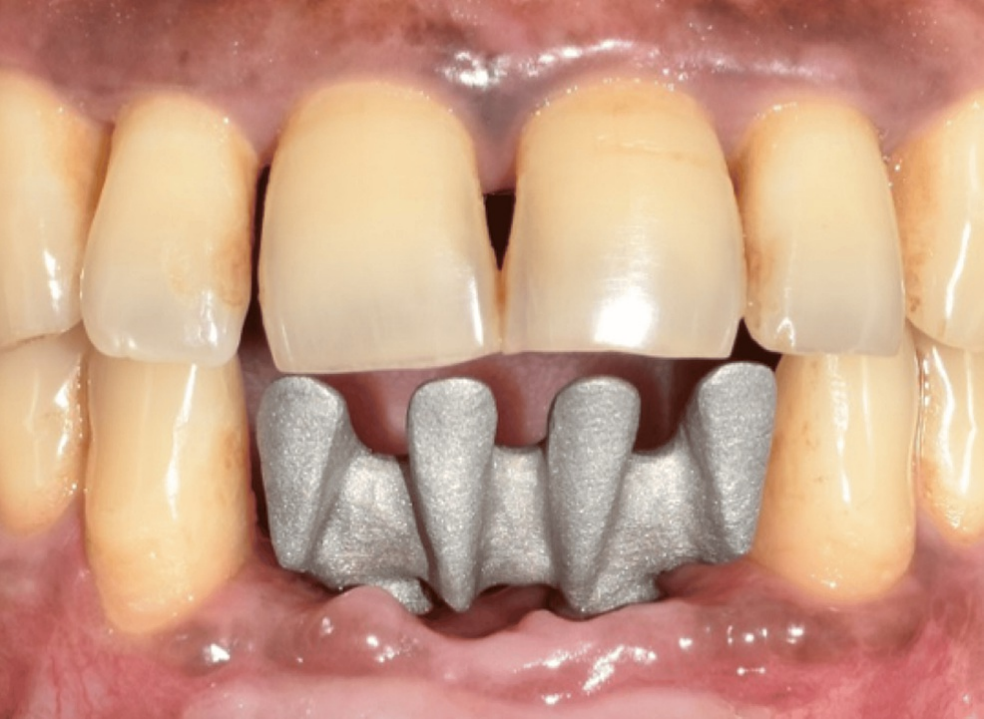
Frontal view of the try-in of the metal framework.

**Figure 19:**
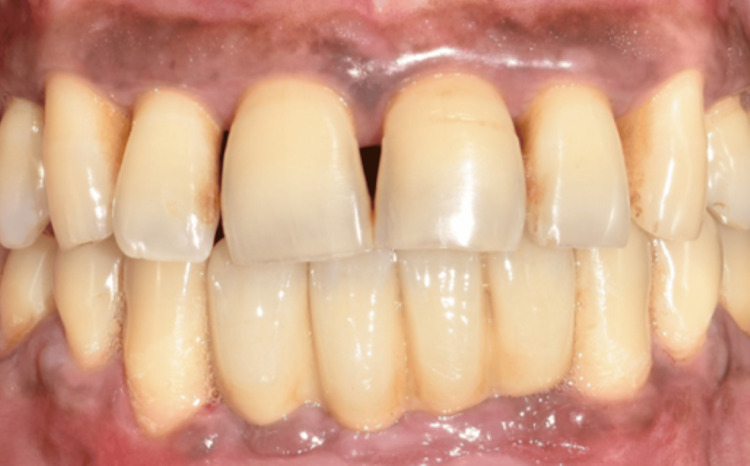
Final implant prosthesis.

The healing abutments were placed after four months of healing. Soft-tissue grafting was performed to enhance the attached gingiva on the labial aspect (Figures 16A-16B). An incision was made to improve gingival contour during the interim prosthesis placement, which was done one month following soft-tissue grafting (Figures 17A-17B). A digital impression was made using implant scan bodies (Figures 17C-17D). This was followed by an intraoral try-in of the metal framework (Figure 18). A screw-retained porcelain fused to a metal implant prosthesis was then delivered (Figure 19). The bone and implant conditions were maintained without any complications at the six-month follow-up appointment.

## Discussion

Dimensional changes to the alveolar ridge following tooth extraction are inevitable. During the healing process, there is a collapse of soft tissue and resorption of the buccal bone, which combine to produce an unpleasant appearance. Several surgical and prosthetic elements influence the final esthetic result. These include tooth extraction, mucosa and buccal bone thickness, 3D implant location, abutment, and provisionalization. Implant angulations in the anterior esthetic zone have to be such that the implants exit crestally, at the proposed cingulum position of the tooth to be replaced [6].

The method used to correct the ridge defects differs according to the degree of resorption. The treatment for horizontal ridge deficiencies involves a variety of techniques, such as sandwich osteotomy combined with sinus floor grafting for severe alveolar atrophy, ridge split, ridge expansion, and guided bone regeneration (GBR). Placing the graft and membrane following decortication may increase the faciolingual bone density. To increase the ridge width, block grafts from extraoral or intraoral sources can also be placed [6].

Autogenous bone grafts, being the only biomaterial that combines the characteristics of osteogenesis, osteoinduction, and osteoconduction, are frequently used in GBR treatments and are regarded as the gold standard. However, drawbacks, including morbidity at the donor site, the sensitivity of the tooth, and the possibility of wound dehiscence have prompted research into the development of bone substitutes for alveolar bone regeneration [7].

Barrier membranes are available in nonresorbable and resorbable forms, and when used to augment bone, they follow the GBR concept. The primary function of GBR is to help maintain the clot and inhibit the migration of connective tissue and epithelial cells into the healing wound, favoring the infiltration of osteogenic cells. The membrane should have good handling qualities, an ideal degradation time, be cell-occlusive, biocompatible, and allow for space maintenance [6,7].

Xenografts have shown excellent characteristics, including biocompatibility, volume maintenance, slow resorption rates, and the ability to form a scaffold. Xenogenous bovine bone substitutes are mostly made of hydroxyapatite following the removal of all organic components similar to the human bone. A biomaterial with particles that have interconnecting pores is formed, enabling the migration of osteoblast cells into the graft and the penetration of new blood vessels [7].

Meloni et al. [8] evaluated the osteoid regeneration in 45 patients with horizontal bone defects using a cohort prospective study. The CBCT scan conducted at the initial examination and seven months post-operatively showed a gain in the crestal bone. The implant success rate was 100% during the follow-up period [8]. Ibrahim et al. [9] evaluated the augmentation of the narrow anterior maxillary alveolar ridge in a randomized clinical trial. There was new bone formation six to seven months postoperatively following xenograft placement.

In both the described cases, there was inadequate keratinized gingiva that needed soft tissue augmentation as it improves peri-implant soft tissue health. Adequate keratinized gingiva aids in plaque control and decreases the risk of marginal bone loss and bleeding on probing [10].

To get the best implant positioning and esthetic outcomes, augmentation was necessary in the cases described. Horizontal bone gain was seen in both cases six months postoperatively. Excellent soft tissue healing occurred, with no occurrence of infections or membrane exposure.

## Conclusions

The process of augmenting a defective ridge is indeed complex and technique-specific. Many augmentation methods and materials have been tried, with positive outcomes. The results of the described cases showed that the sausage technique is a predictable method of horizontal ridge augmentation to achieve appropriate bone volume in the partially edentulous anterior esthetic zones. Diagnosis and treatment planning play a vital role in the successful restoration of implants. The authors believe that a protocol-driven hard and soft tissue augmentation can provide esthetically pleasing and stable long-term results.
